# Supplementary material for: BrainTACO: an explorable multi-scale multi-modal brain transcriptomic and connectivity data resource
Source: Commun Biol. 2024 Jun 14;7:730. doi: 10.1038/s42003-024-06355-7 (PMC11178817; doi:10.1038/s42003-024-06355-7)
Supplement: Supplementary file 2 — Supplementary Information [file 42003_2024_6355_MOESM2_ESM.pdf]

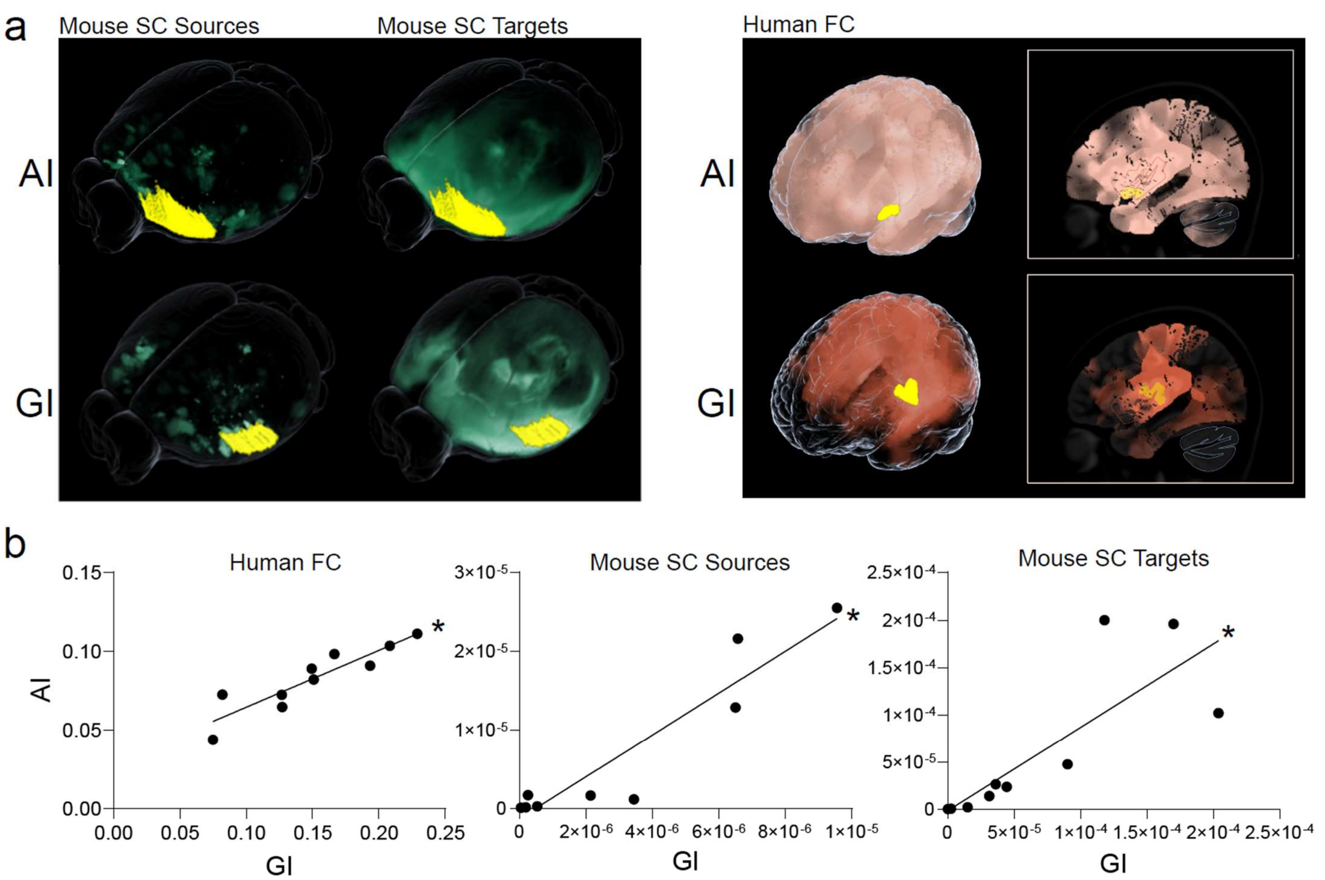


**Supplementary Figure 1. Sampled areas and connectivity analysis of AI and GI in Mouse and Human.** **a.** Mouse area of ”L_Agranular insular area, dorsal part” and ” L_Agranular insular area, ventral part” were combined into AI, while “L_Visceral area” represents GI. Human AI and GI areas were selected by brushing sub-areas within the short and long insular gyri according to https://doi.org/10.1016/j.jhevol.2012.12.003. **b.** Correlations of AI and GI within Human FC (Spearman r = 0.93 , p value = 0.0003) and Mouse SC Source/Targets (Spearman r = 0.92, p value = 0.0005/ Spearman r = 0.94, p value = 0.0002).

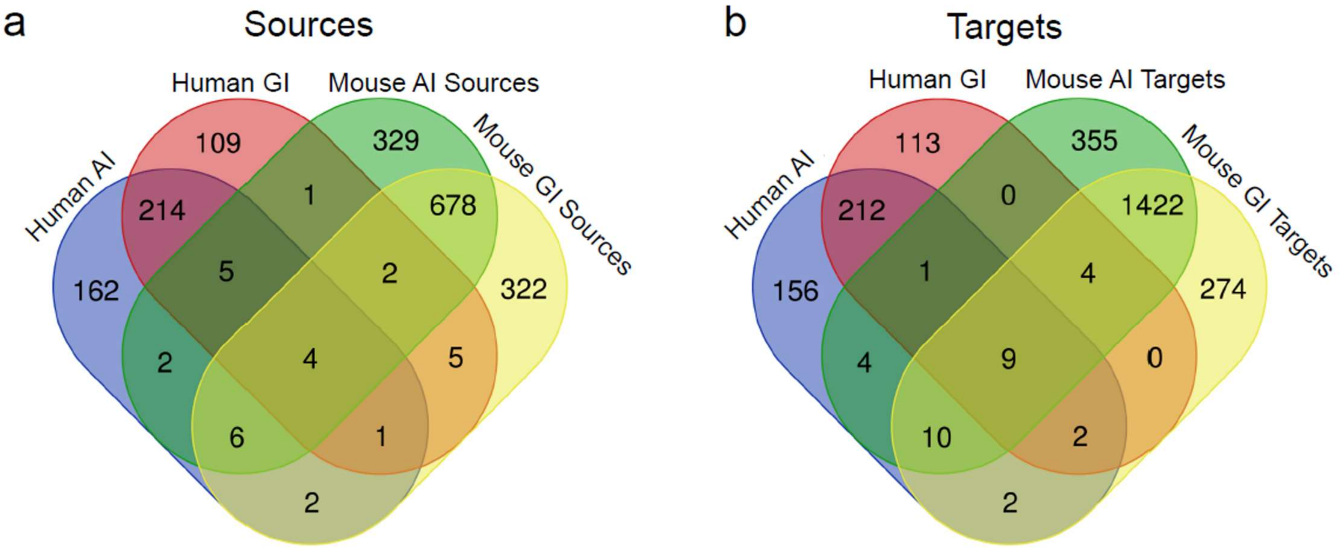


**Supplementary Figure 2. Overlap of top and bottom correlated genes with AI/GI connectivity across human and mouse.**
**a.** Overlap of genes in the top and bottom 1% of gene expression correlations with Human FC and Mouse SC Sources across 10 subcortical areas for AI and GI. **b.** Overlap of genes in the top and bottom 1% of gene expression correlations with Human FC and Mouse SC Targets across 10 subcortical areas for AI and GI. Diagrams were generated with https://bioinformatics.psb.ugent.be/webtools/Venn/ (see Table 3).
