# Supplementary material for: BrainTACO: an explorable multi-scale multi-modal brain transcriptomic and connectivity data resource
Source: Commun Biol. 2024 Jun 14;7:730. doi: 10.1038/s42003-024-06355-7 (PMC11178817; doi:10.1038/s42003-024-06355-7)
Supplement: Supplementary file 3 — Description of Additional Supplementary Files [file 42003_2024_6355_MOESM3_ESM.docx]

**Description of Additional Supplementary Files**

File name: Supplementary Data 1

Description: Mapping and data generation code (related to Figure 1 and 2)

File name: Supplementary Data 2

Description: Dataset comparison code and results (related to Figure 3)

File name: Supplementary Data 3

Description: High resolution version of Figure 4 and 5

File name: Supplementary Data 4

Description: Dataset comparison figure table with additional information, including query brain regions, filters, and cell types (related to Figure 3)

File name: Supplementary Data 5

Description: Consensus hierarchy and cell types (related to Figures 6)

File name: Supplementary Data 6

Description: List of top-bottom 1% genes (related to Figure 6)

File name: Supplementary Data 7

Description: Species overlap (related to Figure 6)

File name: Supplementary Data 8

Description: Association summary (related to Figure 6)
